# Two New Steroidal Saponins from *Ypsilandra thibetica*

**DOI:** 10.1007/s13659-014-0043-1

**Published:** 2014-11-01

**Authors:** Yong-Ai Si, Huan Yan, Wei Ni, Zhen-Hua Liu, Ting-Xiang Lu, Chang-Xiang Cheng, Hai-Yang Liu

**Affiliations:** 1 State Key Laboratory of Phytochemistry and Plant Resources in West China, Kunming Institute of Botany, Chinese Academy of Sciences, Kunming, 650201 China; 2 University of Chinese Academy of Sciences, Beijing, 100049 China

**Keywords:** *Ypsilandra thibetica*, Liliaceae, Ypsilandroside S, Ypsilandroside T

## Abstract

**Abstract:**

Two new monosaccharide steroidal saponins, named ypsilandroside S (**1**) and ypsilandroside T (**2**), have been isolated from the whole plants of *Ypsilandra thibetica*. Their structures were elucidated as heloniogenin 3-*O*-*β*-D-apiofuranoside (**1**) and pregna 5,16-dien-3*β*,12α-diol-20-one-3-*O*-*β*-D-apiofuranoside (**2**) by spectroscopic techniques (1D and 2D NMR, MS). Compounds **1** and **2** were tested for their inhibitory effects on lipopolysaccharide-induced nitric oxide production in RAW 264.7 cells.

**Graphical Abstract:**

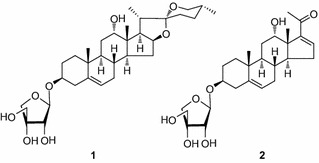

## Introduction

**Fig. 1:**
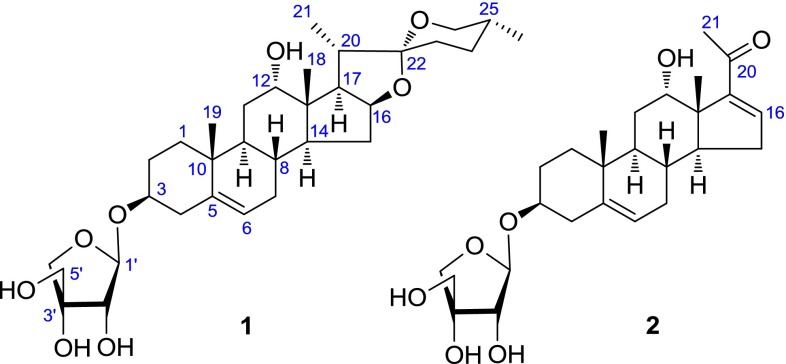
Chemical structures of compounds **1** and **2**

*Ypsilandra* (Liliaceae) is a small genus including only five species and is distributed in southwestern China and Myanmar [1]. *Ypsilandra thibetica* mainly grows in China and has been used in folk medicine for treatment of scrofula, urine negative, edema, uterine bleeding, and traumatic hemorrhage [2, 3]. Our previous investigations suggested that the species is abundant in spirostanol-, furostanol-, and 23-spirocholestanol glycosides with cytotoxic, antifungal, and anti-HIV activities [4–10]. In a continuation of our study on the chemical constituents of this species, we have examined the low polarity part of the EtOH extract of the titled plants. As a result, one new spirostanol saponin, ypsilandroside S (**1**), and one new pregnane glycoside, ypsilandroside T (**2**), were obtained. Herein, we report the isolation, structural elucidation, and anti-inflammatory activities of these two new compounds (Fig. 1).

## Results and Discussion

**Fig. 2:**
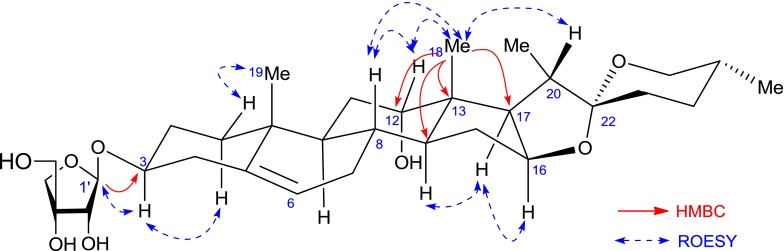
The key HMBC and ROESY correlations of **1**

Compound **1** was obtained as a white, amorphous powder with $\left[ \alpha \right]_{\text{D}}^{ 2 4} \,$ − 78.5 (*c* 0.10, MeOH). Its HR-EI-MS displayed a quasi-molecular ion peak at *m/z* 562.3508 [M]^+^ (calcd. for 562.3506) in accord with the molecular formula C_32_H_50_O_8_, which was confirmed by data from the ^13^C NMR spectrum (Table 1). The IR absorptions at 3441, 2930, and 1631 cm^−1^ implied the existence of OH groups, C=C bonds, and CH groups, respectively. The ^1^H NMR data (Table 1) showed signals for four steroid methyl groups at *δ*_H_ 0.79 (d, *J* = 6.8 Hz), 0.82 (s), 0.97 (d, *J* = 6.8 Hz), 1.02 (s), and an olefinic proton signal at *δ*_H_ 5.38 (br s). The above ^1^H NMR data, together with olefinic carbons signals at *δ*_C_ 141.9 (s, C-5) and 122.7 (d, C-6) and an acetalic carbon signal at *δ*_C_ 110.5 (s, C-22) in the ^13^C NMR spectrum, indicated **1** to be a Δ^5,6^-spirostanol skeleton in the aglycone [4–6]. Comparison of the NMR data of compound **1** with those of ypsilandroside E [5] suggested that they had the same heloniogenin aglycone, which nomenclature is (25*R*)-spirost-5-en-3β,12α-diol [11]. The HMBC and ROESY correlations (Fig. 2) of the aglycone of **1** confirmed the above deduction. Furthermore, the *R*-configuration of C-25 was affirmed by the intensity of the absorptions (899 > 921 cm^−1^) in its IR spectrum [12]. The major difference in sugar moiety between compound **1** and ypsilandroside E were compound **1** only had a pentose and the disappearance of two rhamnopyranosyls in ypsilandroside E. The pentose was elucidated as *β*-D-apiofuranoside by the ^13^C NMR signals at *δ*_C_ 108.5 (d, C-1′), 78.1 (d, C-2′), 80.2 (s, C-3′), 74.6 (t, C-4′), and 65.4 (t, C-5′) with those of the corresponding carbons of *α*- and *β*-D-apiofuranoside and *α*- and *β*-L-apiofuranoside [13, 14]. The HMBC correlations from *δ*_H_ 5.06 (H-1′) to *δ*_C_ 78.8 (C-3) revealed that the sugar chain was attached to C-3. Therefore, the structure of compound **1** was established as heloniogenin 3-*O*-*β*-D-apiofuranoside and given the name ypsilandroside S.

**Table 1:** ^1^H and ^13^C NMR Data for compounds **1** and **2** (*δ* in ppm, *J* in Hz)

No.	**1** ^**a**^		**2** ^**b**^		No.	**1** ^**a**^		**2** ^**b**^	
	*δ*_C_	*δ*_H_	*δ*_C_	*δ*_H_		*δ*_C_	*δ*_H_	*δ*_C_	*δ*_H_
1	38.2 t	1.80 m	37.7 t	1.70 m	18	17.5 q	0.82 s	17.7 q	1.05 s
		1.09 m		1.03 d (3.6)	19	19.6 q	1.02 s	19.7 q	0.98 s
2	30.8 t	1.71 m	30.7 t	2.08 m	20	43.0 d	1.86 m	197.3 s	
		1.40 m		1.69 m	21	14.6 q	0.97 d (6.8)	27.6 q	2.22 s
3	78.8 d	3.40 m	77.9 d	3.74 m	22	110.5 s			
4	39.9 t	2.39 m	39.8 t	2.56 t (2.4)	23	32.4 t	1.71 m		
		2.19 t (12.0)		2.40 m	24	29.4 t	1.65 m (2H)		
5	141.9 s		141.9 s						
6	122.7 d	5.38 br s	122.1 d	5.33 t (5.4)	25	32.8 d	1.58 m		
7	32.6 t	1.69 m	32.3 t	1.96 m	26	67.8 t	3.44 m		
		1.32 m		1.68 m			3.35 m		
8	31.4 d	1.70 m	31.0 d	1.68 m	27	17.5 q	0.79 d (6.8)		
9	45.2 d	1.39 m	46.4 d	1.66 m	Api				
10	37.7 s		37.3 s		1′	108.5 d	5.06 d (2.8)	108.9 d	5.78 d (3.0)
11	29.9 t	1.67 m (2H)	29.6 t	1.82 m (2H)	2′	78.1 d	3.81 d (2.8)	78.4 d	4.77 d (3.0)
					3′	80.2 s		80.7 s	
12	72.9 d	3.67 br s	70.0 d	4.99 t (2.7)	4′	74.6 t	3.97 d (8.8)	75.3 t	4.64 d (9.6)
13	45.6 s		52.6 s				3.72 d (8.8)		4.39 d (9.6)
14	49.1 d	1.67 m	47.6 d	2.58 m	5′	65.4 t	3.57 d (12.0)	65.8 t	4.24 d (11.9)
15	33.0 t	2.01 m	32.4 t	2.29 m			3.54 d (12.0)		4.20 d (11.9)
		1.58 m		2.26 m					
16	81.8 d	4.35 dd (15.0, 7.6)	145.2 d	6.64 dd (3.0, 1.8)					
17	54.5 d	2.48 dd (7.6, 6.8)	154.5 s						

^a^Recorded at 400 MHz in CD_3_OD

^b^Recorded at 600 MHz in C_5_D_5_N

Compound **2** obtained as a white, amorphous powder and had a molecular formula of C_26_H_38_O_7_ on the ground of the HR-EI-MS at *m/z* 462.2599 [M]^+^ (calcd. for C_26_H_38_O_7_ 462.2618) and ^13^C NMR data (Table 1). The ^1^H NMR (Table 1) spectrum of **2** exhibited two tertiary methyls at *δ*_H_ 0.98 and 1.05, and a methyl singlet at *δ*_H_ 2.22 attached to a deshielding moiety, as well as one anomeric proton signal at *δ*_H_ 5.78 (d, *J* = 3.0 Hz). Its IR (1646 cm^−1^), UV [236 nm (log *ε* 4.00)], and ^13^C NMR signals at *δ*_C_ 145.2 (d, C-16), 154.5 (s, C-17), and 197.3 (s, C-20) suggested that compound **2** contained an *α*,*β*-unsaturated carbonyl group. Detailed comparison of the aglycone of compound **2** with those of ypsilandroside R [8] indicated the presence of a hydroxyl group (*δ*_C_ 70.0) instead of a carbonyl group at C-12 in the latter, which was confirmed by the HMBC correlations between the proton signal at *δ*_H_ 1.05 (Me-18) and the carbon signals at *δ*_C_ 70.0 (d, C-12), 52.6 (s, C-13), 47.6 (d, C-14), and 154.5 (s, C-17). The OH-12 was assigned as α-oriented based on the ROESY correlations of H-12 with Me-18. Analysis of the NMR data (Table 1) for the sugar portion of **2** and comparison with those of **1** revealed that they had the same saccharide chain linked at C-3. This was confirmed by the HMBC correlations from *δ*_H_ 5.78 (H-1′) to *δ*_C_ 77.9 (C-3). Consequently, the structure of **2** was elucidated as pregna 5,16-dien-3*β*,12α-diol-20-one-3-*O*-*β*-D-apiofuranoside and named as ypsilandroside T.

Compounds **1** and **2** were rare steroidal saponins with an apiofuranosyl unit directly connecting at C-3 of the aglycone. They were evaluated for their inhibitory effects on the release of NO from macrophages using lipopolysaccharide (LPS)-induced RAW 264.7 cells a model system. The results showed that these two new compounds were inactive with IC_50_ values over 25 μM.

## Experimental Section

### Plant Material

The plant material of *Y. thibetica* were collected in November 2006 from Luding County, Sichuan Province, China, and identified by Prof. Xin-Qi Chen, Institute of Botany, Chinese Academy of Sciences, Beijing. A voucher specimen (No. HY0002) was deposited at State Key Laboratory of Phytochemistry and Plant Resources in West China, Kunming Institute of Botany.

### General Experimental Procedures

Optical rotations were measured on a Jasco P-1020 digital polarimeter. IR spectra were obtained on Bruker Tensor-27 infrared spectrophotometer with KBr pellets. UV spectra were obtained on a Shimadzu UV-2401PC spectrophotometer. ESI-MS and HREI-MS data were obtained with Bruker HTC/Esquire and API Qstar Pulsar mass spectrometers. NMR experiments were performed on Bruker AM-400 and Avance III 600 instrument with TMS as the internal standard. Chemical shifts (*δ*) were expressed in ppm with reference to the solvent signals. Column chromatography (CC) was performed on YWD-3F macroporous resin, silica gel (200–300 mesh, Qingdao Marine Chemical Co., China), and Rp-18 (40–63 μm, Merck). TLC was performed on HSGF_254_ (0.2 mm, Qingdao Marine Chemical Co., China) or Rp-18 F_254_ (0.25 mm, Merck). Fractions were monitored by TLC and spots were visualized by heating silica gel plates sprayed with 10 % H_2_SO_4_ in EtOH. Semi-preparative HPLC was run on Agilent 1100 liquid chromatograph with diode array detector (DAD), Zorbax-SB-C18 column (5 μm; 25 cm × 9.4 mm i.d).

### Extraction and Isolation

The air-dried whole plants of *Y. thibetica* (10 kg) were extracted three times with 70 % EtOH (50 L × 3) under reflux for a total of 6 h and the combined extract was concentrated under reduced pressure. Then the concentrated extract was loaded onto a macroporous resin column (YWD-3F) and eluted successively with H_2_O, 35, 70, and 90 % EtOH, respectively. The 70 % EtOH fraction (70 g) was chromatographed on a silica gel column with a CHCl_3_-MeOH-H_2_O gradient (10:1:0 → 7:3:0.5, v/v) to obtained four fractions. Fr. 1 (20 g) was purified over Rp-18 gel (MPLC, MeOH-H_2_O 5:5 → 9:1) and semi-preparative HPLC (MeCN-H_2_O 20:80 → 40:60 v/v; flow rate: 3 mL min^−1^) to yield **1** (10 mg) and **2** (6 mg).

### Determination of NO Production

RAW 264.7 cells were placed in 96-well plates (2 × 10^5^ cells/well) containing RPMI 1640 medium (Hyclone) with 10 % FBS under a humidified atmosphere of 5 % CO_2_ at 37 °C. After 24 h incubation, cells were treated with the compounds with the maximum concentration of 50 μM in the presence of 1 μg/mL LPS for 18 h. Each compound was dissolved in DMSO and further diluted in cell culture media to obtain different concentrations. NO production was assessed by adding 100 μL of Griess reagent (1 % sulfanilamide and 0.1 % naphthylethylene diaminedihydrochloride in 5 % H_3_PO_4_) to 100 μL supernatant from LPS or the compound-treated cells in triplicate. After 5 min incubation, the absorbance was measured at 570 nm with a 2104 Envision Multilabel PlateReader (Perkin-Elmer Life Sciences, Inc.). MG132 (Sigma Aldrich, purity ≥ 99 %, IC_50_ value = 0.1 μM) was used as a positive control.

### Ypsilandroside S (**1**)

White amorphous powder; $\left[ \alpha \right]_{\text{D}}^{ 2 4} \,$ − 78.5 (*c* 0.10, MeOH); IR (KBr) νmax 3441, 2950, 2930, 2871, 1709, 1631, 1456, 1383, 1300, 1242, 1157, 1096, 1079, 1053, 1009, 981, 959, 921, 899 cm^−1^ (intensity: 899 > 921); ^1^H and ^13^C NMR data see Table 1; positive ESI-MS: *m/z* 585 [M + Na]^+^; HR-EI-MS: *m/z* 562.3508 ([M]^+^, C_32_H_50_O_8_^+^; calcd. 562.3506).

### Ypsilandroside T (**2**)

White amorphous powder; $\left[ \alpha \right]_{\text{D}}^{ 2 4} \,$ − 63.8 (*c* 0.10, MeOH); UV (MeOH) *λ*_max_ (log *ε*) 236 (4.00) nm; IR (KBr) ν_max_ 3441, 2927, 2857, 1646, 1383, 1239, 1097, 1057, 920 cm^−1^; ^1^H and ^13^C NMR data see Table 1; positive-ion ESI-MS: *m/z* 485 [M + Na]^+^; HR-EI-MS: *m/z* 462.2599 ([M]^+^, C_26_H_38_O_7_^+^, calcd. 462.2618).
